# Neuromuscular Rehabilitation of the Brachioradialis Muscle after Distal Radius Fracture in Two Professional Soccer Players Using Electromyographic Biofeedback

**DOI:** 10.3390/muscles3010003

**Published:** 2024-01-23

**Authors:** Verónica Morales-Sánchez, Rafael E. Reigal, Verónica García-Morales, Antonio Hernández-Mendo, Coral Falcó

**Affiliations:** 1 Department of Social Psychology, Social Anthropology, Social Work and Social Services, University of Málaga, 29071 Málaga, Spain; vomorales@uma.es (V.M.-S.); mendo@uma.es (A.H.-M.); 2 Cabra Health Center “Midwife Antonia Mesa Fernández”, 14940 Cabra, Spain; 3 Department of Sport, Food and Natural Sciences, Western Norway University of Applied Sciences, 5020 Bergen, Norway

**Keywords:** electromyographic biofeedback, rehabilitation, neuromuscular rehabilitation, soccer

## Abstract

The use of electromyographic biofeedback (EMG-BF) in the rehabilitation of injuries has been widely referenced in the psychological literature. However, despite some pioneering work in the field of sports, its use in the rehabilitation of sports injuries has hardly been explored. A case of two professional soccer players who each suffered a distal radius fracture is presented here. Parallel to the rehabilitation plan established by medical services, an intervention strategy using EMG-BF was established. An EMG-BF intervention was performed on the brachioradialis muscle with the aim of improving the voluntary control of its electromyographic activity. The study protocol was registered with the identifier NCT05376072. An ABA design was used. In each session, a pre- and postline was recorded to determine the EMG gain acquired at each point of the session. After six sessions, the intervention was terminated. One more follow-up session was performed. The results obtained indicated the efficacy of the intervention; a statistically significant increase in muscle activity in the brachioradialis muscle was observed.

## 1. Introduction

Biofeedback (BF) is a technique by which real-time information on the various physiological parameters of a person’s body can be obtained [1]. BF systems are diverse, but fundamentally, they are based on procedures that transform physiological signals into visual, auditory or tactile information that can be easily interpreted during the performance of a task [2]. In this way, it is possible to observe indirectly how an organ or body system is responding, visualizing various parameters and being able to voluntarily modify the way the organ or system behaves [3,4]. Therefore, it is a very useful resource for the self-regulation of behavior, being a suitable technique for functional rehabilitation and the rehabilitation of injuries [5,6].

Particularly, electromyographic biofeedback (EMG-BF) is a BF technique based on electromyographic (EMG) procedures, which allow the electrical activity of skeletal muscles to be recorded and displayed in real time [7,8]. When muscle activation is high, the signal amplitude provided is intense, and when the activation decreases, the signal amplitude is milder. Thus, EMG-BF allows a user to receive information about his or her muscle functioning and to interact with the EMG-BF [9,10]. Thanks to this, by means of EMG-BF, people can increase their perceptions of muscle activation and relaxation states, increasing their awareness about changes in their tonicity and improving their voluntary control [7]. There are different procedures by which this technique can be applied, one of the most widely used being surface EMG-BF [11,12]. Specifically, this is a noninvasive procedure in which electrodes are attached to the surface of the muscle, allowing its EMG activity to be recorded and providing information during physical exercise or functional activity [13,14].

Since EMG-BF improves the perception of skeletal muscle activity and a person’s learning to voluntarily regulate its contractile capacity, its implementation is considered useful and, in several cases, has been effective in rehabilitation processes after muscle dysfunction events due to stroke or other pathologies, as well as after osteoarticular and muscle injuries caused in the sports context [8,12,15,16]. As an example, the efficacy of EMG-BF for the functional recovery of the quadriceps in patients who have undergone surgery to reconstruct the anterior cruciate ligament [17] and after meniscus repair by arthroscopy [18] has been highlighted. Its efficacy has also been observed in improving strength in the flexor muscles of the fingers of people with chronic pain and muscle weakness [19]. Likewise, EMG-BF has been found to be effective in improving the motor function of the paretic upper limb during the subacute stage following stroke [12]. 

Contact sports have a high injury rate; one such contact sport is soccer, and osteoarticular and muscular problems occur frequently in this discipline [20]. In relation to this example, previous evidence has indicated that EMG-BF can be a useful procedure for applications in the context of soccer, improving the expectations of functional recovery and the readaptation to sports activity after various types of injuries [21,22]. Bone, joint and muscle problems in the lower limbs are more prevalent in soccer [23,24]. However, collisions and tripping during play expose the upper limbs not only to direct trauma but also to indirect trauma during falls, which can lead to injuries of various kinds [25,26]. 

There is evidence that 30% of injuries in soccer players occur in the upper extremities, ranging from mild contusions that do not have serious consequences to more severe injuries that require surgical intervention [27]. Among the various trauma injuries that can occur in the upper limbs during soccer practice is radius fracture [28,29]. In soccer, distal radius fractures, which can be with bone displacement or without displacement, are frequent due to falls or contusions [25,30]. If the fractures are complex and cannot be solved by orthopedic procedures, they are likely to require surgical interventions, which compromises recovery time and requires more specific care and measures to prevent relapses [31].

In addition, the decision to perform surgery takes into consideration the patient’s age, the type of patient, the risk of new bone displacement, the type of activity, etc. [32]. When this solution is chosen, the most frequent surgical interventions to resolve a distal radius fracture are based on invasive procedures to reduce bone displacement: by fixation with stainless steel or titanium pins, using plates and screws or with external fixators [33,34]. In these cases, the incision caused by surgery and the mechanical procedure to reduce bone displacement could compromise the musculotendinous structure and its functionality [34]. Moreover, this situation can decrease the confidence to carry out certain contact actions [28,35]. The brachioradialis, along with certain other muscles, is special in this regard because it is inserted into the radius itself, and so radial injury can have effects on the force exerted during elbow flexion or on wrist functionality after surgery and immobilization [36,37]. 

Procedures such as EMG-BF could be effective in recovering the contractile capacity of a muscle and in providing better voluntary control of its activity [38]. In fact, EMG-BF has been widely used in the recovery from muscle injuries in soccer [21,22]. However, this type of injury is less frequent in soccer than in other sports, so no data have been reported on the use of EMG-BF in this situation. However, the use of the upper extremities is essential for actions such as jumping, protecting yourself from falls or having the space to play [28,35]. Therefore, the aim of this study was to explore the efficacy and effectiveness of EMG-BF treatment in two soccer players who had suffered a displaced distal radius fracture and had undergone surgery in which pins had been used for fixation and the reduction in bone displacement. This paper presents a study of two cases and aims to meet the efficacy criteria described by Chambless and Holon [39]. Both cases were expected to achieve a functional capacity like the uninjured hemilateral limb.

## 2. Materials and Methods

### 2.1. Design

This research is a case study using a quasiexperimental within-subject manipulative design (A → B → A) with pretest (baseline) and post-test evaluations. The study protocol is registered at https://www.clinicaltrials.gov/ (accessed on 29 April 2022) with the identifier NCT05376072. There are several procedures that can be used when biofeedback is applied. The protocol used in this research conforms to the so-called BFB (biofeedback) training [40], in which a person voluntarily modifies the activity of his or her musculature following knowledge of the electrophysiological signal being emitted during previous contractions. 

### 2.2. Participants

The sample for this study included two professional soccer players from the Second Division of the Spanish Soccer League. The first was a 21-year-old male, weighing 78 kg and 180 cm tall. He had a diagnosis of left distal radius fracture on the dominant side with bone displacement and had undergone surgery for bone pinning two weeks prior to the present study. The second was a 24-year-old male, weighing 77 kg and 179 cm tall. He had a diagnosis of right distal radius fracture on the dominant side with bone displacement and had undergone surgery for bone pinning three weeks prior to the present study. In both cases, before the biofeedback treatment, they realized functional recovery exercises and were treated with laser therapy. At the direction of the club’s medical services, they received daily physical rehabilitation sessions.

#### Measurements and Instruments

The electromyographic data were recorded by using an 8-channel ProComp Infiniti biofeedback unit from Thought Technology, which has a sampling rate of between 256 Sa/s and 2048 Sa/s. This unit is composed of a Decoder Unit and a TT-USB Interface Unit connected by a fiber optic cable. An electrode was placed on a MyoScan-Pro unit. The placement was performed following the muscle distribution of the lesion. The placement in the muscle was carried out parallel to the muscle fibers and in the upper part of the crest of the muscle with the arm horizontal to the ground. Muscle preparation before electrode placement consisted of cleaning the area and removing body hair for a smooth surface.

### 2.3. Procedure

In order to carry out the research, the intervention procedure was explained to the participants, who then signed an informed consent form after agreeing to take part in the study. Permission was also requested from the club and the medical services that were assisting the players. How the research would be carried out was explained to them in detail, and they were informed that the data would be treated anonymously and that they could withdraw at any time if they wished to do so. In addition, the principles promulgated in the Helsinki Declaration [41] were respected, and this study was approved by the Ethics Committee of the University of Malaga (CEUMA, no. 243, 19-2015-H).

The intervention program lasted for fifteen days (Table 1). Eight work sessions and seven rest sessions (one between each work session) were carried out. The two players performed the first session of ten trials, which served as a baseline. The average electromyographic activity was evaluated during isometric contraction at maximum effort in both brachioradialis muscles, recording the amplitude of the signal in microvolts. Each trial lasted six seconds, and there was a two-minute rest period between trials. In total, this session lasted approximately 25 min. The maximum intensity reached by the noninjured hemilateral limb was the one used as a target for the injured limb to work toward during the intervention program.

Subsequently, and after a rest day, the two soccer players underwent six sessions with EMG-BF on alternate days, one day of work and another of rest. These sessions were divided into three phases: (a) three baseline trials without receiving feedback; (b) six trials with biofeedback on electromyographic activity, in which they performed isometric contractions of the left or right brachioradialis muscle as appropriate; and (c) three trials without feedback. In phases “a” and “c”, the same procedure as the pretreatment session was performed. Each trial lasted six seconds, and there was a two-minute rest period between trials. In total, this session lasted approximately 30 min. To ensure that the electrodes were always in the same place, they were marked with ink every day.

Finally, in the eighth session, ten trials in which the efficacy of the intervention was tested were performed again. For this session, we used the maximum and mean electromyographic activity values (amplitude measured in microvolts), which were calculated during the time of maintained muscle tension during each trial. Each trial lasted six seconds, and there was a two-minute rest period between trials. In total, this session lasted approximately 25 min.

**Figure 1:**
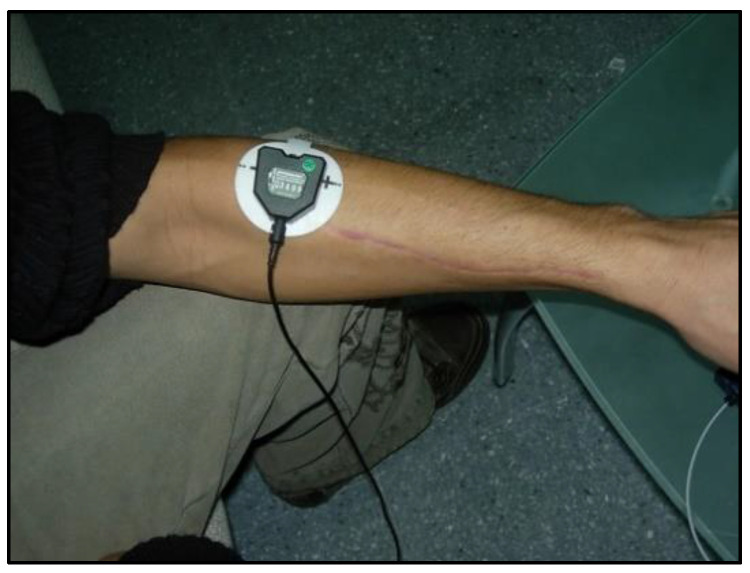
Electrode placement and arm position.

In all the trials, they performed isometric contractions of the brachioradialis muscle. To perform the contractions, the participants were seated with the upper limb extended in front and parallel to the ground. The elbow was extended, the fist was closed and the wrist was extended (Figure 1). During the isometric contraction, the amplitude, the mean and the peak of the electromyographic signal, as well as the contraction and stiffening times, were recorded. The contraction time was defined as the duration between the initiation of the contraction and the moment when the targeted muscle activity is attained. Conversely, the stiffening time denotes the interval during which the attained tension is sustained [42]. For this work, the maximum and mean electromyographic activity values obtained during the period of muscle contraction were considered.

**Figure 2:**
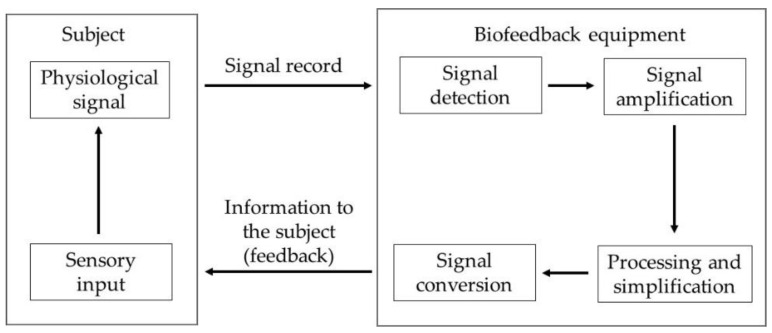
Architecture and operation of biofeedback system.

During the exercise with EMG-BF, the system showed visual signals through a display in which the user could see a continuous line over time, which rose or fell depending on the intensity of the contraction. In addition, the system displayed a value in microvolts that corresponded to the visual evolution of the line. Figure 2 shows the architecture and operation of the biofeedback system.

### 2.4. Data Analysis 

The information collected by the EMG-BF system was processed with BioGraph Infiniti software v2 (Thought Technology Ltd., Montreal, QC, Canada). From it, descriptive and inferential analyses were performed. The mean, standard deviation, skewness and kurtosis of the study variables were calculated. The Shapiro–Wilk statistic was also calculated to determine the normality of the data. If all these tests (skewness, kurtosis and Shapiro–Wilk test) indicated that the data showed normality, we used the ANOVA test. If not, we used nonparametric tests. A two-way repeated-measures ANOVA was performed to determine the differences in the mean and maximum values (amplitude in microvolts) between session 1 and 8. The Wilcoxon test was used to evaluate the differences in the mean and maximum values (amplitude in microvolts) between trials with biofeedback and without biofeedback. The effect size was calculated with the eta squared and Cohen’s d statistic (≈0.20: small, ≈0.50: medium and ≈0.80: large [43]). SPSS Statistics v.24 software (IBM Corp., Armonk, NY, USA) was used for most of the statistical analyses. SAS v.9.1 software (SAS Institute Inc., Cary, NC, USA) was used for the analysis of variance components [44,45], and SAGT v.1.0 software (University of Malaga, Malaga, Spain) was used for the generalizability analysis [46]. An analysis of the variance components was performed by using the least squares strategy (VARCOMP Type 1), which is based on decomposing the total variance into related components, and the maximum likelihood strategy (GLM), which is based on seeking values in the model factors that make the observed data more probable [47,48]. A generalizability analysis is a procedure that, after scrutinizing the sources of variation affecting a measurement, provides an estimate of how well the observed mean aligns with the mean of all possible observations. In this analysis, the relative G coefficient is calculated as a measure of reliability, and the absolute G coefficient is calculated as a measure of generalizability [46].

## 3. Results

### 3.1. Analysis of Variance Components

A variance component analysis (Table 2) was performed by using a five-facet model for the maximum EMG signal (y = p f s e n) and for the mean EMG signal (z = p f s e n), where p = participant, f = phase, s = session, e = trial and n = trial number. Initially, these models were used without interactions because of the saturation generated by the inclusion of so many facets.

In both models, a least square (VARCOMP Type1) and a maximum likelihood (GLM) strategy were used to check whether the error variances were equal in both procedures and thus to ensure that the sample was linear, normal and homoscedastic [46,47]. It was possible to verify that in both the first model (y = p f s e n) and the second model (z = p f s e n), the error variance was equal by using both procedures. Thus, for the model (y = p f s e n), the error variance was VARCOMP Type1 = 277,879 and GLM = 277,879.241, and for the model (z = p f s e n), it was VARCOMP Type1 = 162,062 and GLM = 162,062.1620. It is therefore assumed that the sample for both models had a linear, normal and homoscedastic distribution. 

The model (y = p f s e n) was also found to be significant and explained 75% of the variance. In addition, all facets, except for n (trial number), were significant. Similarly, the model (z = p f s e n) was significant and explained 75% of the variance. In addition, all facets, with the exception of n (number of trials), were significant.

Subsequently, two four-facet models with interaction were proposed: (1) the (y = p|f|s|e) model of maximum signal estimation and (2) the (z = p|f|s|e) model of mean signal estimation, where p = participant, f = phase, s = session and e = trial.

It could be verified, as in the previous case, that in both models ((y = p|f|s|e) and (z = p|f|s|e)), the error variance was the same in both procedures. Thus, for the first model, the error variance was VARCOMP Type1= 109,013 and GLM = 109,012.651, and for the second model, it was VARCOMP Type1 = 69,253 and GLM = 69,252.9820. It is therefore assumed that the sample for both models had a linear, normal and homoscedastic distribution.

The model (y = p|p|f|s|e) was significant and explained 90% of the variance. In addition, all facets were significant. All interactions were also significant except for (f|s), (p|f|s), (f|s|e) and (p|f|s|e), which collapsed due to the contribution of p (participant), f (phase) and s (session).

As with the previous model, the model (z = p|f|s|e) was significant and explained 89% of the variance. Similarly, all facets were significant. All interactions were also significant, except for (f|s), (p|f|s), (f|s|e) and (p|f|s|e), which collapsed due to the contribution of p (participant), f (phase) and s (session).

#### Generalizability Analysis

From the variance component analysis, and using the sum of squares, a generalizability analysis was implemented for the two models, (y = p|f|s|e) and (z = p|f|s|e). A four cross-facet analysis was carried out for the two models, where each facet was used sequentially as an instrumentation facet.

For the model of maximum signals, (y = p|f|s|e), a previous analysis of variance was performed, which revealed that the highest percentages of variance associated with each of the facets were (p) (participant), 26.83%; (f) (phase), 24.17%; (s) (session), 10.34%; (e) (trial), 10.74%; and 14.94% for the (p)(s) interaction (Table 3). 

The results show that the relative and absolute G indices (reliability and generalizability, respectively) were excellent. The lowest value (0.661) corresponded to the model (f) (s) (e)/(p), where (p) acts as the instrumentation facet (the facet to be estimated) and facets (f), (s) and (e) act as differentiation facets. This value can be explained by considering that facet (p) has the highest percentage of variance associated with it.

In relation to the model of mean signals, (z = p|f|s|e), a previous analysis of variance was performed, which revealed that the highest percentages of variance associated with each of the facets were (p) (participant), 19.62%; (f) (phase), 31.91%; (s) (session), 11.14%; (e) (trial), 5.54%; and 10.48% for the (p) (f) interaction and 14.34% for the (p) (s) interaction (Table 4).

The results show that for all the models, the relative and absolute G indices (reliability and generalizability, respectively) were excellent. The lowest corresponding values were 0.675, 0.776 and 0.781; the first two corresponded to the model (f) (s) (e)/(p), and the third corresponded to the model (s) (e) (p)/(f), with (p) and (f) acting as instrumentation facets (the facet to be estimated) and the facets (f) (s) (e) and (s) (e) (p) acting as differentiation facets. These values can be explained by considering that facets (p) and (f) have the highest percentages of associated variance.

### 3.2. Differences in Maximum and Mean Values between Trials with BF and Trials without BF

Table 5 shows the maximum and mean values of electromyographic activity for the six intervention sessions (from sessions 2 to 7) for the pre- and postfeedback trials as well as for the biofeedback trials.

The Wilcoxon test was performed to determine the differences between trials with biofeedback and trials without biofeedback. The results indicated statistically significant differences in both maximum (Z = −6.72 *p* < 0.001; Cohen’s d = 1.02, 95% CI (0.53, 1.51)) and mean (Z = −6.58; *p* < 0.001; Cohen’s d = 0.817, 95% CI (0.34, 1.29)) values.

**Figure 3:**
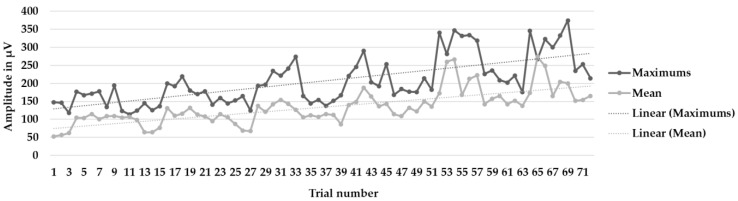
Learning curves during the six intervention program sessions. Mean and maximum amplitude values, as well as the trendlines (linear), in the 72 trials with biofeedback and without biofeedback.

**Figure 4:**
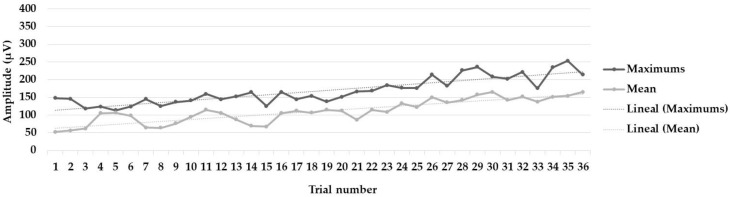
Learning curves during the six intervention program sessions. Mean and maximum amplitude values, as well as the trendlines (linear), in the 36 trials without biofeedback.

**Figure 5:**
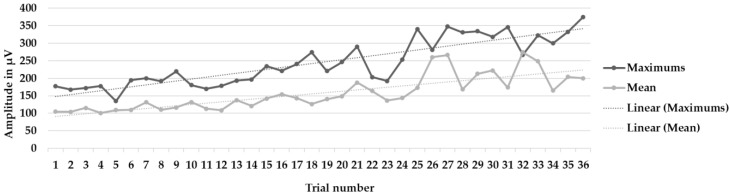
Learning curves during the six intervention program sessions. Mean and maximum amplitude values, as well as the trendlines (linear), in the 36 trials with biofeedback.

Figure 3, Figure 4 and Figure 5 show the learning curves during the six intervention program sessions. Figure 3 displays the mean and maximum amplitude values in the trials with biofeedback and without biofeedback (72 trials). Figure 4 and Figure 5 show the mean and maximum values during the trials only, without biofeedback and with biofeedback, respectively (36 trials in each figure).

### 3.3. Differences in Electromyographic Activity between Session 1 and Session 8

Table 6 shows the maximum and mean values of electromyographic activity for sessions 1 and 8. It is evident that there was an increase in electromyographic activity after the biofeedback intervention.

To determine whether the differences between session 1 and 8 were significant, a two-way repeated-measures ANOVA was performed. The results showed statistically significant differences for both the maximum (F_[1,39]_ = 78.04, *p* < 0.001, η^2^ = 0.67) and mean values (F_[1,39]_ = 198.18, *p* < 0.001, η^2^ = 0.74).

## 4. Discussion

The aim of this research was to explore the effects of an EMG-BF intervention on the control of muscle activity in the brachioradialis muscle of the forearm after a distal radius fracture with displacement of the radius and after undergoing surgery in two professional soccer players. The results obtained reveal the efficacy of the treatment, with a significant increase in the electrophysiological activity of the muscle under study.

First, the results obtained show statistically significant differences in brachioradialis muscle electromyographic activity between trials with biofeedback and trials without biofeedback. This suggests that the information provided to soccer players during their exertion-condition muscle-contraction performance is congruent with previous studies indicating that EMG-BF improves voluntary muscle regulation [7,8,9,10]. Furthermore, the differences found suggest that this is a procedure that provides useful, fast and effective information, given that, within a single work session, it causes a significant behavioral change in muscle activation [9,10].

Second, the data obtained indicate that the interventions during six sessions produced a statistically significant improvement in muscle activity between the preintervention session and the last session, both with and without biofeedback. Furthermore, the observed effect size is high, reflecting the efficacy of the treatment. These results suggest that the intervention with EMG-BF led to learning in the regulation of brachioradialis muscle behavior, given that both its mean and maximum values experienced a substantial increase in its electromyographic activity. The effect described in this research is congruent with the results found in previous studies, which have highlighted how EMG-BF has proved to be an adequate procedure for improving the muscle dysfunction caused by different pathologies [8,12,15,16,17,18,19]. Moreover, it has shown its usefulness in resolving muscular affectations derived from injuries in the sports field, specifically in soccer [20,21]. 

Our findings contribute to highlighting the efficacy of EMG-BF in the functional recovery of skeletal musculature after injury, which has important practical implications. First, the immobilization of a body segment due to bone injury leads to a loss of muscle mass and weakening of its contractile capacity [36,37]. Thus, implementing this type of procedure facilitates the recovery of muscle tone and the ability to self-regulate its activity. This helps the athlete to readapt more quickly, and with greater confidence, to competitive situations, reducing the possibility of relapse and difficulties in sports performance [28,35,38]. In addition, it is a technique that causes the rapid and high-level learning of the contractile capacity of the muscle. This suggests that it can be used as a complement to other functional readaptation treatments that are prescribed to reinforce the success of an intervention and, thus, the recovery of the athlete. 

The analysis of variance indicates that the model is significant and explains 75% of the variance. This, together with the high effect size found, shows that the intervention carried out has a high explanatory value and represents a technique that has a great capacity to modify the voluntary behavior of the muscle under study. Furthermore, the generalizability analyses show that the data presented in this study are reliable and can be generalized, which increases the value of the present study as it can be extrapolated to the population as a whole.

This research has some limitations. First, the sample is small. Although a generalizability analysis was performed, it would be interesting to carry out other studies on different and larger samples to verify that the findings are corroborated. Second, there is no follow-up evaluation of the results obtained. Although it was observed that rapid and robust learning occurs with the EMG-BF procedure, it would be appropriate to analyze whether, and to what extent, this is maintained over time. Third, EMG-BF has been used in the context of soccer for the retraining of electromyographic activity in certain muscles. However, it would be interesting to increase the evidence from other muscles to determine the efficacy of EMG-BF in different injuries.

Nevertheless, the results obtained show that the EMG-BF procedure used for intervention on the brachioradialis muscle after a distal radius fracture was effective in improving the voluntary activity of the muscle in the cases analyzed. Furthermore, the effect size was high, which suggests the weight that this type of technique can have in the recovery process of an athlete. Thus, EMG-BF could be a suitable tool for use in the recovery processes of athletes, increasing the possibility of achieving better recovery and better rehabilitation of the damaged area.

## 5. Conclusions

These findings highlight that BF-EMG could be considered an effective tool, along with other therapeutic procedures, in muscle-recovery processes after distal radius fracture. Specifically, it could contribute to improving the muscle tone of the brachioradialis muscle when this type of fracture occurs, reducing the impact that inactivity could have on the adjacent muscles. Specifically, it has been observed that a fifteen-day program was effective in improving the contractile capacity of the brachioradialis muscle, which suggests that BF-EMG has positive effects on the recovery of this muscle in a short period of time.

## Figures and Tables

**Table 1:** Timeline of the sessions and trials.

Day	1	2	3	4	5	6	7	8	9	10	11	12	13	14	15
Session	(T)	(R)	(T)	(R)	(T)	(R)	(T)	(R)	(T)	(R)	(T)	(R)	(T)	(R)	(T)
	10		12		12		12		12		12		12		10
	trials		trials		trials		trials		trials		trials		trials		final trials
	baseline		a/b/c		a/b/c		a/b/c		a/b/c		a/b/c		a/b/c		

Note. (T) = training day; (R) = rest day; a/b/c = (a) three baseline trials without receiving feedback; (b) six trials with biofeedback on electromyographic activity, in which they performed isometric contractions of the left or right brachioradialis muscle as appropriate; and (c) three trials without feedback.

**Table 2:** Analysis of variance components.

**Model z = Means-EMG**	**Sources of Variation**	**Sum of Squares**	**Degrees of Freedom**	**Mean Square**	**F**	**Pr < F**	**%**
	(p)	99,005.04	1	99,005.04	157.26	<0.0001	19.62
	(f)	192,112.50	2	96,056.25	152.57	<0.0001	31.91
	(p)(f)	27,258.95	2	13,629.47	21.65	<0.0001	10.48
	(s)	118,500.80	5	23,700.16	37.64	<0.0001	11.14
	(p)(s)	46,684.39	4	11,671.10	18.54	<0.0001	14.34
	(f)(s)	0	0	0	0	0	0
	(p)(f)(s)	0	0	0	0	0	0
	(e)	50,256.65	9	5584.07	8.87	<0.0001	5.54
	(p)(e)	6982.46	9	775.83	1.23	0.2828	1.21
	(f)(e)	11,859.62	10	1185.96	1.88	0.0549	1.88
	(p)(f)(e)	3159.98	10	315.99	0.50	0.8855	1.36
	(s)(e)	8340.64	5	1668.12	2.65	0.0266	0.41
	(p)(s)(e)	5965.22	4	1491.30	2.37	0.0570	2.06
	(f)(s)(e)	0	0	0	0	0	0
	(p)(f)(s)(s)(e)	0	0	0	0	0	0
**Model y = Maximums-EMG**	(p)	206,481.35	1	206,481.35	208.35	<0.0001	26.83
	(f)	234,004.41	2	117,002.20	118.06	<0.0001	24.17
	(p)(f)	15,756.65	2	7878.33	7.95	0.0006	3.35
	(s)	201,490.42	5	40,298.08	40.66	<0.0001	10.34
	(p)(s)	84,059.18	4	21,014.79	21.21	<0.0001	14.94
	(f)(s)	0	0	0	0	0	0.
	(p)(f)(s)	0	0	0	0	0	0
	(e)	164,282.93	9	18,253.65	18.42	<0.0001	10.74
	(p)(e)	21,378.09	9	2375.34	2.40	0.0161	2.82
	(f)(e)	26,392.10	10	2639.21	2.66	0.0060	2.44
	(p)(f)(e)	6745.96	10	674.59	0.68	0.7403	1.67
	(s)(e)	21,928.59	5	4385.72	4.43	0.0010	1.69
	(p)(s)(e)	4913.24	4	1228.31	1.24	0.2985	0.97
	(f)(s)(e)	0	0	0	0	0	0
	(p)(f)(s)(s)(e)	0	0	0	0	0	0

**Table 3:** Results of the generalizability analysis of the model (y = p|f|s|e).

Face	Levels	Size Universe	Description	Variance	Model Generalizability	G Relative	G Absolute
(p)	2	INF	participants	26.832	(f) (s) (e)/(p)	0.806	0.661
(f)	3	INF	phases	24.170	(s) (e) (p)/(f)	0.965	0.866
(s)	6	INF	session	10.340	(p) (f) (e)/(s)	0.961	0.939
(e)	10	INF	test	10.745	(p) (f) (s)/(e)	0.988	0.975

Note. INF = infinity.

**Table 4:** Results of the generalizability analysis of the model (y = p|f|s|e).

Face	Levels	Size Universe	Description	Variance	Model Generalizability	G Relative	G Absolute
(p)	2	INF	participants	19.624	(f) (s) (e)/(p)	0.776	0.675
(f)	3	INF	phases	31.918	(s) (e) (p)/(f)	0.922	0.781
(s)	6	INF	session	11.148	(p) (f) (e)/(s)	0.963	0.939
(e)	10	INF	test	5.547	(p) (f) (s)/(e)	0.992	0.986

Note. INF = infinity.

**Table 5:** Descriptive statistics for maximum and mean values of electromyographic activity for trials with and without biofeedback, as well as pre- and post-BF.

Electromyographic Activity (µV)						
Values	Testing	M	SD	S	K	S-W
Maximums	Without biofeedback	158.53	48.97	1.61	2.98	0.85 ***
	With biofeedback	227.80	82.86	1.44	2.01	0.86 ***
Mean	Without biofeedback	103.67	38.69	0.72	1.14	0.93 **
	With biofeedback	144.58	59.36	2.57	2.21	0.70 ***

Note. M = mean; SD = standard deviation; S = skewness; K = kurtosis; S-W = Shapiro–Wilk; µV = microvolts. ** *p* < 0.01; *** *p* < 0.001.

**Table 6:** Maximum and mean values of electromyographic activity for sessions 1 and 8.

Electromyographic Activity (µV)						
Values	Session	M	SD	S	K	S-W
Maximums	1	99.62	21.18	−0.48	−0.97	0.92
	8	247.24	71.66	0.55	−1.10	0.93
Mean	1	48.85	10.27	0.12	−1.04	0.95
	8	186.63	58.35	0.24	−1.51	0.91

Note. M = mean; SD = standard deviation; S = skewness; K = kurtosis; S-W = Shapiro–Wilk; µV = microvolts.

## Data Availability

The data presented in this study are available upon request from the corresponding author.
